# Examination of the Suitability of Vericiguat in Non-Heart Failure with Preserved Ejection Fraction Patients with Improved Ejection Fraction

**DOI:** 10.3390/jcm13175264

**Published:** 2024-09-05

**Authors:** Haruyuki Kinoshita, Hiroshi Sugino, Kento Fujita, Yoji Sumimoto, Kenji Masada, Takashi Shimonaga, Akiyo Suga, Mayumi Toko, Kaori Taniyasu, Saki Ushirozako, Yumiko Katayama, Chiemi Hirahara, Masahiro Takada

**Affiliations:** 1 Department of Cardiology, NHO Kure Medical Center, Kure 737-0023, Japan; sugino.hiroshi.mj@mail.hosp.go.jp (H.S.); hujita.kento.sg@mail.hosp.go.jp (K.F.); sumimoto.yoji.wn@mail.hosp.go.jp (Y.S.); masada.kenji.sr@mail.hosp.go.jp (K.M.); shimonaga.takashi.vb@mail.hosp.go.jp (T.S.); 2 The Ultrasound Team, Physiological Examination Department, NHO Kure Medical Center, Kure 737-0023, Japan; suga.akiyo.gd@mail.hosp.go.jp (A.S.); toko.mayumi.de@mail.hosp.go.jp (M.T.); taniyasu.kaori.hq@mail.hosp.go.jp (K.T.); nagoshi.saki.ru@mail.hosp.go.jp (S.U.); katayama.yumiko.nk@mail.hosp.go.jp (Y.K.); hirahara.chiemi.zc@mail.hosp.go.jp (C.H.); 3 Department of Pharmacy, NHO Kure Medical Center, Kure 737-0023, Japan; takada.masahiro.gu@mail.hosp.go.jp

**Keywords:** improved EF, Four Pillars, quintuple therapy, worsening heart failure, Fantastic Four

## Abstract

**Background/Objectives**: Vericiguat has been shown to reduce cardiovascular mortality and hospitalisation for heart failure in patients with reduced ejection fraction. While Vericiguat is considered one of the standard treatments for heart failure, it is unclear under which conditions Vericiguat would be most effective. With a focus on the prognosis and improved EF of heart failure, we aimed to investigate in which cases Vericiguat is suitable for use in addition to standard cardioprotective drugs. **Methods**: We prospectively compared echocardiograms taken before and after the administration of Vericiguat in 46 patients with non-dialysis and without heart failure with preserved ejection fraction (non-HFpEF) (left ventricle ejection fraction [LVEF] < 50%) who were able to continue Vericiguat in addition to other standard heart failure drugs (the “Fantastic Four”) for more than 6 months at our hospital. Patients who showed an improvement of 10 points or more in LVEF were defined as improved EF+. **Results**: LVEF improved significantly from 38 [33–45]% at the time of administration to 46 [35–54.5]% at 6 months (*p* < 0.001). When comparing patients with and without improved EF, a significant difference was observed in the Hb (OR = 1.66, 95%CI = 1.12–2.83, *p* = 0.028), early introduction (OR = 12.5, 95%CI = 1.58–149, *p* = 0.025), and initiation of Vericiguat after the administration of the Fantastic Four (OR = 9.79, 95%CI = 1.71–100.2, *p* = 0.022). **Conclusions**: In this study, the early administration of Vericiguat, haemoglobin value, and initiation of Vericiguat after the introduction of the Fantastic Four were identified as independent factors for eligibility in non-dialysis, non-HFpEF patients who were able to continue GDMT treatment for more than 6 months after adding Vericiguat.

## 1. Introduction

It has been reported that the prognosis of patients with heart failure improves when the left ventricular ejection fraction (LVEF) is improved [1]. Now that evidence is available for angiotensin receptor/neprilysin inhibitors and sodium glucose cotransporter-2 inhibitors (SGLT-2 inhibitors), reports of quadruple medical therapy (also called the Four Pillars [2] or Fantastic Four) have been increasing [3], and multidrug therapy for heart failure has become essential and standard.

Although great expectations are now being placed on quintuple therapy including Vericiguat [4] and the ESC and AHA/ACC guidelines recommend Vericiguat as Class IIb for HFrEF [5,6], there are several restrictions on the administration of Vericiguat [7,8]. As such, it is thought that its introduction may lag behind that of the other cardioprotective drugs known as the Four Pillars in the United States and Europe. On the other hand, in Japan, Vericiguat is only available to patients who are receiving standard treatment for chronic heart failure, so the door seems to be open. However, even in Japan, Vericiguat is not introduced early on but rather in circumstances unfavourable for heart failure, such as when a worsening heart failure (WHF) has occurred, a rehospitalisation for heart failure, or in stage C or D heart failure [9]. As a result, it seems that the drug’s true potential is not being fully utilised. A subanalysis of the VICTORIA trial showed that Vericiguat is expected to reduce cardiovascular death and rehospitalisation rates in high-risk heart failure patients, partly due to significant improvements in left ventricular structure and function [10]. Moreover, it is unclear in which types of cases Vericiguat is suitable, even though it is listed as part of quintuple therapy.

This study investigated which cases are more suitable for Vericiguat administration by focusing on the prognosis of heart failure and improved EF.

## 2. Methods

**Figure 1:**
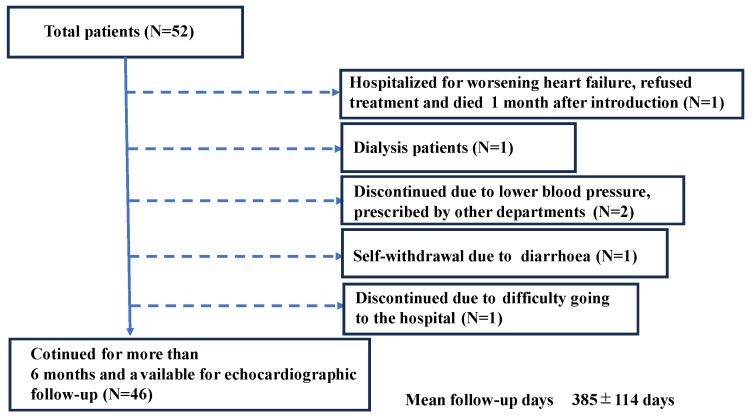
Illustration of the study protocol. Vericiguat was administered to a total of 52 non-dialysis, non-HFpEF patients. Six patients were excluded: one was a patient who was hospitalised with worsening heart failure and refused to eat or take medication due to cognitive decline and subsequently died; one was a dialysis patient; two were discontinued after being transferred to other departments; one self-withdrew due to diarrhoea; and one was discontinued because he was unable to be followed up at our hospital.

This prospective study included patients who received Vericiguat between November 2022 and December 2023. Safety analyses were performed on all patients. Efficacy analyses were performed on patients who visited our hospital after continuing Vericiguat for 6 months or more. We enrolled non-HFpEF patients who were able to continue taking Vericiguat for more than six months after its initiation. We excluded patients who could not continue taking Vericiguat for more than six months due to the progression of dementia or their own judgment; had difficulty visiting the hospital; or died or, for some reason, were unable to undergo a cardiac ultrasound or blood sampling tests six months later. In addition, non-HFpEF patients who were undergoing dialysis were not included in this study (Figure 1).

This study was conducted in accordance with the Declaration of Helsinki and was approved in advance by our hospital’s ethical review board (ethics review board number 2023-43). Consent was obtained from patients due to the prospective and opt-out nature of this study. Patients had a history of heart failure but were non-dialysis and without heart failure with preserved ejection fraction (non-HFpEF) (left ventricle ejection fraction [LVEF] < 50%). Guideline-based medication was administered at the discretion of the treating cardiologist, and Vericiguat was initiated during outpatient care or hospitalisation for first-time heart failure. Vericiguat dosage started at 2.5 mg and was titrated every 1 or 3 months to a target dose of 10 mg. If patients had hypotensive symptoms such as dizziness or lightheadedness, the dose was reduced or titration was postponed. Baseline characteristics were collected from participants, including laboratory, echocardiographic, and medication data. Echocardiographic data were measured by an experienced, certified echocardiographer (Japanese Society of Echocardiography) independently to the attending physician, and LVEF was assessed using 2D echocardiography and compared before and 6 months after the administration of Vericiguat. Regarding improvement in LVEF: an improvement of 10 points or more in LVEF has been reported to be most strongly correlated with the prognosis [11], so the group in which LVEF improved by 10 points or more 6 months after Vericiguat administration compared to the time of initiation was defined as “improved EF+”. There are various definitions for when the onset of heart failure occurs [9,12], but here, in cases of acute heart failure or acute exacerbation of chronic heart failure, the time of onset of heart failure was defined as the date of first hospitalisation for heart failure. However, in cases of chronic heart failure, the time of onset was defined as the date when structural abnormalities were recognised in the heart and diuretics or standard heart failure medications were started. In 46 patients who continued Vericiguat treatment for more than 6 months, a logistic regression analysis was used to compare the “improved EF+” and “improved EF-” groups before starting and at 6 months. Statistical evaluation utilised JMP pro ver9.0 (SAS Institute Inc). Variables with *p* < 0.05 were considered significant. Continuous data were expressed as mean and standard deviation or median and group comparisons used *t*-tests, Wilcoxon rank–sum tests, and Wilcoxon signed-rank tests. Comparisons of categorical data used the chi-square test or Fisher’s exact test, as appropriate.

## 3. Results

**Figure 2:**
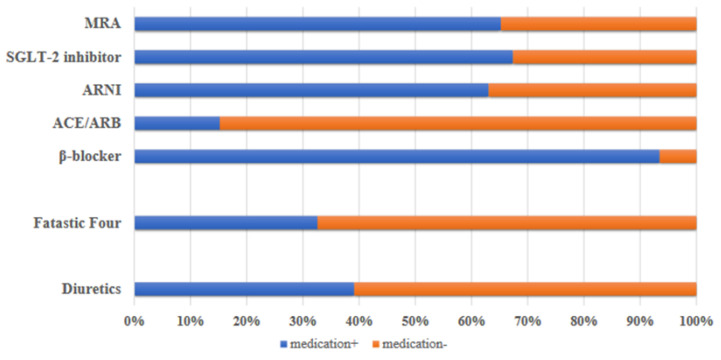
Prescription rates of standard heart failure medications in 46 patients. At the time of initiation of Vericiguat, the use rate of ARNI was over 60%, while that of MRA and SGLT-2 inhibitors was just under 70%. The use rate of beta-blockers was over 90%.

Of the 55 patients who received Vericiguat, 46 had data including echocardiography after 6 months (3 were transferred to a different department or hospital or gave up due to low blood pressure, feeling unwell, etc.) (Figure 1). The mean age was 76.2 ± 12.4 years, and 39 participants were male (Table 1). A total of 36 patients (78%) received renin-angiotensin system inhibitors, 29 of whom received Sacubitril/Valsartan. A total of 43 patients (93%) received beta-blockers, and 30 (65%) received mineralocorticoid receptor antagonists. A total of 31 patients (67%) received SGLT-2 inhibitors. A total of 18 patients (39%) received diuretics such as lupus diuretics and Tolvaptan (Figure 2). 

**Figure 3:**
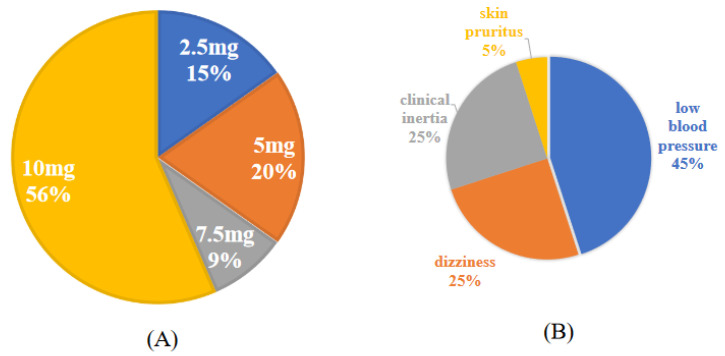
Details of the capacity of the administered Vericiguat (**A**) and details of why the dose did not reach 10 mg (**B**). The target dose of Vericiguat was 10 mg, but in 44% of cases (**A**), the target dose could not be reached. The main reasons for this were fatigue caused by low blood pressure and dizziness. In prescription cases at other hospitals, there were also cases where the dosage was continued as it was when the prescription was requested (**B**).

At the end of the study, 26 patients (56%) were taking 10 mg Vericiguat, 4 patients (9%) were taking 7.5 mg, 9 patients (20%) were taking 5 mg, and 7 patients (15%) were taking 2.5 mg. Dose reduction or discontinuation was at the discretion of the attending cardiologist depending on blood pressure and subjective symptoms. Vericiguat was administered for a mean duration of 385 days. The median maintenance dose was 10 mg [5,10] (Figure 3).

**Figure 4:**
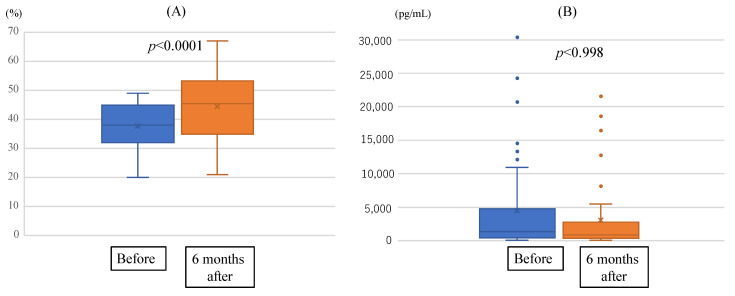
Changes in LVEF (**A**) and NT-proBNP (**B**) before and six months after Vericiguat administration. Comparing pre- and 6-month Vericiguat treatments, there was a significant improvement in LVEF, but no statistical improvement was shown for NT-proBNP.

The median left ventricular ejection fraction (LVEF) was 38% (32%, 45%) before administration of Vericiguat, and 45% at 6 months after introduction (*p* = 0.188) (Figure 4A). The median N-terminal fragment of brain natriuretic peptide precursor (NT-proBNP) was 1342 (447, 4760) pg/mL at baseline and 892 (352, 2803) pg/mL at 6 months after administration of Vericiguat (*p* = 0.188) (Figure 4B). 

Both LVEF and NT-proBNP were not normally distributed, so the Wilcoxon signed-rank test was performed. 

Logistic regression analysis performed on groups with and without improved EF identified three independent factors:

1. Haemoglobin levels;
2. Administer all four Fantastic Four drugs first, then add Vericiguat;
3. Early administration of Vericiguat is indicative of suitability for the introduction of Vericiguat (Table 2).

## 4. Discussion

**Figure 5:**
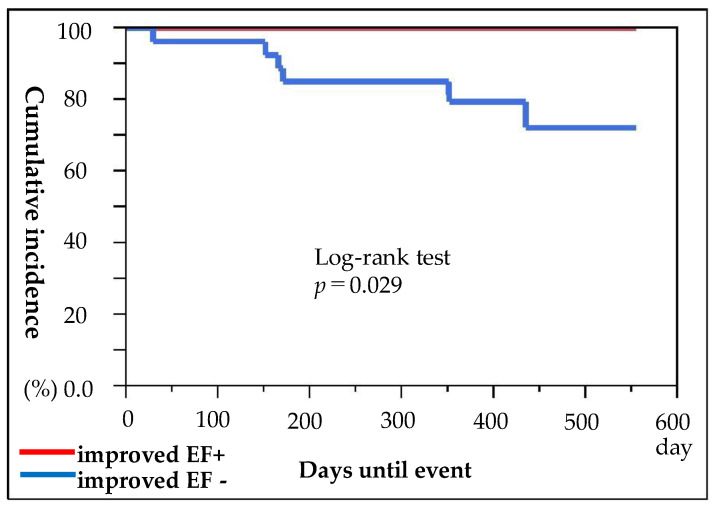
Estimates of the Cumulative Incidence of the first cardiovascular death or hospitalisation due to heart failure. Comparing improved EF+ and improved EF- at 6 months after starting Vericiguat treatment, the risk of first cardiovascular death or hospitalisation due to heart failure was significantly lower in improved EF+ patients.

Vericiguat, a novel orally soluble guanylate stimulator, enhances the cyclic guanosine monophosphate pathway by directly stimulating soluble guanylate cyclase, independent of nitric oxide. In addition, Vericiguat sensitises soluble guanylate cyclase to endogenous nitric oxide by stabilising the nitric oxide binding site [13,14]. The VICTORIA trial demonstrated the effects of Vericiguat therapy to reduce cardiovascular mortality and heart failure hospitalisations compared with placebo in patients with heart failure with reduced ejection fraction (HFrEF) who were at high risk for worsening heart failure [14,15]. For various reasons, not all patients in this study reached a Vericiguat dose of 10 mg, but there were, nonetheless, significant improvements in actual prognosis (Figure 5). 

In a subanalysis of the VICTORIA trial, no significant difference was found in the effects of Vericiguat across quartiles on haemoglobin values, but the cutoff for the first quartile of haemoglobin was 12.1 g/dL or less, which is a different range to the one used in this study. In comparison with the first quartile of haemoglobin, higher haemoglobin corresponded to better patient outcome [16], so we infer that it is necessary to pay close attention to haemoglobin values when introducing Vericiguat.

As several studies have reported that the standard combination of cardioprotective drugs, the Fantastic Four, can be expected to produce beneficial prognoses [17,18], it is recommended that HFrEF patients be treated with this combination regimen as much as possible [19]. In addition, a randomised controlled trial on the efficacy and safety of early introduction of standard treatment drugs showed significant outcomes in the intensive treatment group (i.e., the group in which guideline-directed medical therapy [GDMT] was introduced early and up-titrated early) [20].

In this study, early introduction was defined as the administration of Vericiguat within 100 days of the onset of heart failure. This is based on the SOCRATES-REDUCED study, which observed a significant improvement in LVEF in the group in which Vericiguat was increased from 2.5 mg to 10 mg at 12 weeks [21]. Furthermore, since the approximate maximum interval between outpatient visits after improvement in a patient’s heart failure is three months, there are psychological hurdles for medical professionals to start a new oral medication after that time [22]. In addition, patients may be financially and psychologically reluctant to take more oral medication if their heart failure has stabilised; thus, we defined early introduction as initiating Vericiguat within 100 days of the onset of heart failure.

Since the effectiveness of Vericiguat has not been demonstrated in cases where NT-proBNP levels have become extremely high [15], it may be necessary to consider introducing Vericiguat before it becomes “too late”. Furthermore, there are reports stating that there is an obvious benefit to starting Vericiguat early [23], and we consider there to be no reason for postponement in cases where Vericiguat can be introduced.

This study investigated which cases are more suitable for Vericiguat, acting as “a brace (in Japanese, sujikai) supporting all or any of the Four Pillars” among patients with heart failure receiving therapy based on the four standard cardioprotective drugs, which include not only MRAs, beta-blockers, and Sacubitril/Valsartan but also SGLT2 inhibitors, in actual clinical practice in Japan.

## 5. Limitation

This study was a prospective study with a very small sample size. It was conducted at a single facility, and the median observation period was 385 days. Due to the small sample size, some differences of clinical significance may not have reached statistical significance. As all standard heart failure medications, including the Four Pillars were continued along with Vericiguat for more than 6 months, the influence of medications other than Vericiguat on the improvement in LVEF cannot be excluded. Therefore, this study investigated what circumstances were necessary for an improvement of 10 or more points in LVEF in combination with Vericiguat. In addition, since we only examined cases in which Vericiguat was continued for more than 6 months, and cases in which Vericiguat had to be discontinued and patients who died soon after its introduction were not included, it is necessary to continue to pay attention to patient trends in the early stages of its introduction.

## 6. Conclusions

A significant improvement in left ventricular ejection fraction was observed in non-dialysis, non-HFpEF patients when Vericiguat was administered in addition to standard heart failure medications. Particularly, in non-dialysis, non-HFpEF patients, the following three points should be considered to improve LVEF by ≥10 points when Vericiguat is added to standard heart failure medications as follows: (1) adjustment of haemoglobin levels; (2) administer all four Fantastic Four drugs first, then add Vericiguat; and (3) early administration of Vericiguat.

## Figures and Tables

**Table 1:** Baseline characteristics of patients in the non-dialysis and non-HFpEF group with introduction of Vericiguat.

	N = 46
Mean age (SD)-yr	76.2 (12.4)
man-no. (%)	39 (85)
CAD-no. (%)	30 (65)
CAF-no. (%)	13 (28)
DM-no. (%)	23 (50)
HT-no. (%)	33 (72)
LVEF (Q1, Q3)-%	38 (32, 45)
NYHA-no. (%)	
I	3 (7)
II	23 (50)
III	19 (41)
IV	1 (2)
NT-proBNP (Q1, Q3)-pg/mL	1342 (447, 4760)
Hb mean (SD)-g/dL	12.4 (2.2)
BUN (Q1, Q3)-mg/dL	21.5 (16.4–26.5)
eGFR mean (SD)-mL/min/1.73 m^2^)	44.1 (17.5)
number of hospitalization for Heart failure (Q1, Q3)-no	1 (1, 2)
early administration of Vericiguat-no. (%)	9 (20)

Values are mean (SD), n (%), or median (Q1–Q3). CAD = Coronary Artery Disease, CAF = chronic atrial fibrillation, DM = diabetes mellitus, HT = hypertension, LVEF = left ventricular ejection fraction, NYHA = New York Heart Association functional classification, NT-proBNP = N-terminal pro-brain natriuretic peptide, Hb = haemoglobin, BUN = blood urea nitrogen, eGFR = estimated glomerular filtration rate.

**Table 2:** Stratification by presence or absence of improved EF.

	Univariate Analysis			Multivariate Analysis		
	n = 19	n = 27				
	**Improved EF+**	**Improved EF-**	***p* Value**	**OR**	**95%CI**	***p* Value**
**Mean age (SD)-yr**	78.0 (9.9)	74.9 (13.9)	0.403			
**man-no. (%)**	16 (84)	23 (85)	0.928			
**CAD-no. (%)**	11 (58)	19 (70)	0.382			
**CAF-no. (%)**	6 (32)	7 (26)	0.675			
**DM-no. (%)**	7 (37)	16 (59)	0.134			
**HT-no. (%)**	14(74)	19(70)	0.806			
**LVEF (Q1, Q3)-%**	36 (27, 45)	38 (33, 45)	0.203			
**NT-proBNP (Q1, Q3)-pg/mL**	1309 (415, 4350)	1412 (649, 6986)	0.503			
**Hb mean (SD)-g/dL**	13.3 (2.2)	11.7 (2.1)	0.017	1.66	1.12–2.83	0.028 **☨**
**BUN (Q1, Q3)-mg/dL**	21.2 (15.6, 27.7)	21.7 (18, 26.1)	0.929			
**eGFR mean (SD)-mL/min/1.73 m^2^**	46.2 (18.1)	42.6 (17.2)	0.495			
**MRA-no. (%)**	15 (79)	15 (56)	0.101			
**SGLT-2 inhibitor-no. (%)**	14 (74)	17 (63)	0.445			
**ARNI-no. (%)**	16 (84)	13 (48)	0.013			
**ACE i/ARB-no. (%)**	1 (5)	6 (22)	0.115			
**β-blocker-no. (%)**	18 (95)	25 (93)	0.772			
**Fantastic Four-no. (%)**	10 (53)	5 (19)	0.015	9.79	1.71–100.2	0.022 **☨**
**Diuretics-no. (%)**	4 (21)	14 (52)	0.035	0.28	0.05–1.38	0.134
**early administration of Vericiguat-no. (%)**	6 (32)	3 (11)	0.085	12.5	1.59–148	0.025 **☨**

Values are mean (SD), n (%), or median (Q1, Q3). CAD = Coronary Artery Disease, CAF = chronic atrial fibrillation, DM = diabetes mellitus, HT = hypertension, LVEF = left ventricular ejection fraction, NYHA = New York Heart Association functional classification, NT-proBNP = N-terminal pro-brain natriuretic peptide, Hb = haemoglobin, BUN = blood urea nitrogen, eGFR = estimated glomerular filtration rate, MRA = mineralocorticoid receptor antagonist, ARNI = angiotensin receptor/neprilysin inhibitor, ACE i = angiotensin-converting enzyme inhibitor, ARB = angiotensin receptor blocker, ☨ = significant.

## Data Availability

Data are available from the corresponding author upon reasonable request.

## Abbreviations

CAD	Coronary Artery Disease
CAF	chronic atrial fibrillation
DM	diabetes mellitus
HT	Hypertension
LVEF	left ventricular ejection fraction
NYHA	New York Heart Association functional classification
NT-proBNP	N-terminal pro-brain natriuretic peptide
Hb	Haemoglobin
BUN	blood urea nitrogen
eGFR	estimated glomerular filtration rate
MRA	mineralocorticoid receptor antagonist
ARNI	angiotensin receptor/neprilysin inhibitor
ACE i	angiotensin-converting enzyme inhibitor
ARB	angiotensin receptor blocker
WHF	worsening heart failure
GDMT	guideline-directed medical therapy
improved EF+	The group in which the left ventricular ejection fraction at 6 months after the administration of Vericiguat improved by 10 points or more compared to at the time of introduction
improved EF-	The group in which the improvement in left ventricular ejection fraction did not reach 10 points six months after the start of Vericiguat administration

## References

[1] Savarese G., Vedin O., D’Amario D., Uijl A., Dahlström U., Rosano G., Lam C.S., Lund L.H. (2019). Prevalence and Prognostic Implications of Longitudinal Ejection Fraction Change in Heart Failure. JACC Heart Fail..

[2] Straw S., McGinlay M., Witte K.K. (2021). Four pillars of heart failure: Contemporary pharmacological therapy for heart failure with reduced ejection fraction. Open Heart.

[3] Greene S.J., Khan M.S. (2021). Quadruple Medical Therapy for Heart Failure: Medications Working Together to Provide the Best Care. J. Am. Coll. Cardiol..

[4] Greene S.J., Bauersachs J., Brugts J.J., Ezekowitz J.A., Filippatos G., Gustafsson F., Lam C.S.P., Lund L.H., Mentz R.J., Pieske B. (2023). Management of Worsening Heart Failure With Reduced Ejection Fraction: JACC Focus Seminar 3/3. J. Am. Coll. Cardiol..

[5] McDonagh T.A., Metra M., Adamo M., Gardner R.S., Baumbach A., Böhm M., Burri H., Butler J., Čelutkienė J., Chioncel O. (2022). 2021 ESC Guidelines for the diagnosis and treatment of acute and chronic heart failure: Developed by the Task Force for the diagnosis and treatment of acute and chronic heart failure of the European Society of Cardiology (ESC) With the special contribution of the Heart Failure Association (HFA) of the ESC. Rev. Espanola Cardiol..

[6] Heidenreich P.A., Bozkurt B., Aguilar D., Allen L.A., Byun J.J., Colvin M.M., Deswal A., Drazner M.H., Dunlay S.M., Evers L.R. (2022). 2022 AHA/ACC/HFSA Guideline for the Management of Heart Failure: A Report of the American College of Cardiology/American Heart Association Joint Committee on Clinical Practice Guidelines. Circulation.

[7] FDA Statement on VERQUVO January 2021. https://www.accessdata.fda.gov/drugsatfda_docs/label/2021/214377s000lbl.pdf.

[8] European Medicines Agency Statement on VERQUVO. https://www.ema.europa.eu/en/documents/product-information/verquvo-epar-product-information_en.pdf.

[9] Tsutsui H., Isobe M., Ito H., Okumura K., Ono M., Kitakaze M., Kinugawa K., Kihara Y., Goto Y., Komuro I. (2019). JCS 2017/JHFS 2017 Guideline on Diagnosis and Treatment of Acute and Chronic Heart Failure—Digest Version. Circ. J..

[10] Pieske B., Pieske-Kraigher E., Lam C.S., Melenovský V., Sliwa K., Lopatin Y., Arango J.L., Bahit M.C., O’Connor C.M., Patel M.J. (2023). Effect of vericiguat on left ventricular structure and function in patients with heart failure with reduced ejection fraction: The VICTORIA echocardiographic substudy. Eur. J. Heart Fail..

[11] Aimo A., Fabiani I., Vergaro G., Arzilli C., Chubuchny V., Pasanisi E.M., Petersen C., Poggianti E., Taddei C., Pugliese N.R. (2021). Prognostic value of reverse remodelling criteria in heart failure with reduced or mid-range ejection fraction. ESC Heart Fail..

[12] The Japanese Heart Failure Society’s Recommendation on Heart Failure Using NT-pro BNP 17 November 2023. https://www.asas.or.jp/jhfs/english/outline/guidelines_20180822.html.

[13] Stasch J.-P., Pacher P., Evgenov O.V. (2011). Soluble Guanylate Cyclase as an Emerging Therapeutic Target in Cardiopulmonary Disease. Circulation.

[14] Armstrong P.W., Roessig L., Patel M.J., Anstrom K.J., Butler J., Voors A.A., Lam C.S.P., Ponikowski P., Temple T., Pieske B. (2018). A Multicenter, Randomized, Double-Blind, Placebo-Controlled Trial of the Efficacy and Safety of the Oral Soluble Guanylate Cyclase Stimulator: The VICTORIA Trial. JACC Heart Fail..

[15] Armstrong P.W., Pieske B., Anstrom K.J., Ezekowitz J., Hernandez A.F., Butler J., Lam C.S., Ponikowski P., Voors A.A., Jia G. (2020). Vericiguat in Patients with Heart Failure and Reduced Ejection Fraction. N. Engl. J. Med..

[16] Ezekowitz J.A., Zheng Y., Cohen-Solal A., Melenovský V., Escobedo J., Butler J., Hernandez A.F., Lam C.S.P., O’Connor C.M., Pieske B. (2021). Hemoglobin and Clinical Outcomes in the Vericiguat Global Study in Patients With Heart Failure and Reduced Ejection Fraction (VICTORIA). Circulation.

[17] Vaduganathan M., Claggett B.L., Jhund P.S., Cunningham J.W., Ferreira J.P., Zannad F., Packer M., Fonarow G.C., McMurray J.J.V., Solomon S.D. (2020). Estimating lifetime benefits of comprehensive disease-modifying pharmacological therapies in patients with heart failure with reduced ejection fraction: A comparative analysis of three randomised controlled trials. Lancet.

[18] Tromp J., Ouwerkerk W., van Veldhuisen D.J., Hillege H.L., Richards A.M., van der Meer P., Anand I.S., Lam C.S., Voors A.A. (2022). A Systematic Review and Network Meta-Analysis of Pharmacological Treatment of Heart Failure With Reduced Ejection Fraction. JACC Heart Fail..

[19] Matsukawa R., Okahara A., Tokutome M., Itonaga J., Koga E., Hara A., Kisanuki H., Sada M., Okabe K., Kawai S. (2023). A scoring evaluation for the practical introduction of guideline-directed medical therapy in heart failure patients. ESC Heart Fail..

[20] Mebazaa A., Davison B., Chioncel O., Cohen-Solal A., Diaz R., Filippatos G., Metra M., Ponikowski P., Sliwa K., A Voors A. (2022). Safety, tolerability and efficacy of up-titration of guideline-directed medical therapies for acute heart failure (STRONG-HF): A multinational, open-label, randomised, trial. Lancet.

[21] Gheorghiade M., Greene S.J., Butler J., Filippatos G., Lam C.S., Maggioni A.P., Ponikowski P., Shah S.J., Solomon S.D., Kraigher-Krainer E. (2015). Effect of Vericiguat, a Soluble Guanylate Cyclase Stimulator, on Natriuretic Peptide Levels in Patients With Worsening Chronic Heart Failure and Reduced Ejection Fraction: The SOCRATES-REDUCED Randomized Trial. JAMA.

[22] Akita K., Kohno T., Kohsaka S., Shiraishi Y., Nagatomo Y., Izumi Y., Goda A., Mizuno A., Sawano M., Inohara T. (2017). Current use of guideline-based medical therapy in elderly patients admitted with acute heart failure with reduced ejection fraction and its impact on event-free survival. Int. J. Cardiol..

[23] Rao V.N., Diez J., Gustafsson F., Mentz R.J., Senni M., Jankowska E.A., Bauersachs J. (2023). Practical Patient Care Considerations With Use of Vericiguat After Worsening Heart Failure Events. J. Card. Fail..

